# Summarizing physical performance in professional soccer: development of a new composite index

**DOI:** 10.1038/s41598-024-65581-5

**Published:** 2024-06-24

**Authors:** José M. Oliva-Lozano, Mattia Cefis, Víctor Fortes, Roberto López-Del Campo, Ricardo Resta

**Affiliations:** 1 Health Research Centre, University of Almería, Almería, Spain; 2 Università degli Studi di Brescia, Brescia, Italy; 3 Unión Deportiva Almería, Almería, Spain; 4 Department of Competitions and Mediacoach, LaLiga, Madrid, Spain

**Keywords:** Football, Monitoring, High Intensity, PLS-SEM, Training, Scientific data, Statistics

## Abstract

The aims of this study were to create a composite index to measure the overall players’ physical performance in professional soccer matches and analyze the effect of individual playing time and positional differences on this composite index. A total of 830 official matches from LaLiga men’s first division and Spanish Copa del Rey were analyzed, which resulted in 24,980 match observations collected from 1138 male players (forwards, n = 286; midfielders, n = 441; defenders, n = 411). The physical performance variables, which represent the locomotor demands, were collected using electronic performance tracking systems. A Partial Least-Squares Structural Equation Model (PLS-SEM) was used to measure performance. The PLS-SEM output had three significant latent components, which explained 95% of the initial variability, that were related to the acceleration-specific performance (component 1), high-intensity running-related variables (component 2), and medium intensity actions variables (component 3). Also, a linear regression analysis was used to explore relationships between playing activity time (hours—X axis) and the composite index (10-point scale—Y axis), in which a strong and positive correlation was observed between individual playing time and the composite index (*r* = 0.76; *p* < 0.001; R^2^ = 0.58). Also, significant positive correlations were observed in forwards (*r* = 0.85; *p* < 0.001; R^2^ = 0.74), midfielders (*r* = 0.80; *p* < 0.001; R^2^ = 0.64), and defenders (*r* = 0.67; *p* < 0.001; R^2^ = 0.45). However, significant differences between playing positions with a small effect size (*p* < 0.05; eta-squared = 0.01) were found. From a practical perspective, this study may serve as a reference for sports performance practitioners to create a composite index that measures the overall players’ physical performance. The instructions to create this index are available in the manuscript.

## Introduction

Soccer stands as the most widely embraced sport across the globe, engaging millions of participants worldwide, although only a select few transition to the professional level^1^. This team sport is typified by an intermittent activity pattern, involving high-intensity anaerobic actions interspersed with periods of reduced intensity^2,3^. Soccer matches impose the most significant load in a single session within the weekly training regimen and coaches typically take this into account when structuring the weekly training plan^4^. Consequently, it’s imperative for sports performance and medical professionals to assess physical performance to ensure that players are adequately prepared for the demands of competition^5^.

In recent years, the adoption of electronic performance and tracking systems (EPTS) has allowed professionals to gain a deeper understanding of the physical demands in soccer^6,7^. This data can be collected for both matches and training sessions^8^. These tracking systems encompass camera-based technologies and wearable devices, incorporating a mix of positioning systems (e.g., Global Positioning Systems or Local Positioning Systems), inertial measurement units (e.g., accelerometers, gyroscopes, and magnetometers), and physiological monitors (e.g., heart rate monitors)^6,7^. Specifically, EPTS allow the gathering of external load (e.g., distance covered, accelerations, decelerations, or sprints) and internal load (e.g., psychophysiological responses like mean or peak heart rate) data^2,6,7^. Therefore, these tools are invaluable to professionals since the information obtained from monitoring players during competition informs decisions not only related to the training schedule and but also during a session as well (e.g., live performance data)^9^.

Nonetheless, managing data and interpreting variables pose significant challenges for sports performance and medical experts using EPTS^10,11^. Practitioners often receive daily physical performance reports containing approximately from 100 to 200 variables^10^. As a result, there is a need to condense these large datasets, requiring practitioners to employ appropriate methods to pinpoint and select key performance indicators after each session’s data collection^10,11^. Considering that one of the major hurdles for sports performance and medical practitioners using EPTS is the handling of data and the interpretation of numerous variables^2,11^, the summarization of extensive data is necessary. Professionals must apply appropriate methods to pinpoint and select key performance indicators once data has been collected in each session^2,11^. In this regard, resent research suggested the use of Partial Least-Squares Structural Equation Model (PLS-SEM) to measure performance^12,13^. Utilizing freely accessible key performance indicators sourced from sofifa.com, previous research developed composite indicators for mobile players in the top 5 European Leagues using a Third Order PLS-SEM model, albeit without considering physical performance^12^. PLS-SEM, overall, provides a flexible (i.e., being a non-parametric tool) and robust method for analyzing composite indicators, rendering it a valuable resource for researchers in diverse domains such as social sciences, management, and economics^12^.

However, when measuring performance using PLS-SEM, the effect of playing time and playing position should be taken into account. The reason is that differences in playing time between matches (e.g., differences in match duration due to extra time or 30-min overtime periods)^14,15^, players’ participation in match-play (e.g., starters vs non-starters)^16,17^, and physical demands by playing position^18,19^ may be observed.

Therefore, the aims of this study were to (1) create a composite index to measure the overall players’ physical performance in professional soccer matches and (2) analyze the effect of individual playing time and positional differences on the new index. The hypotheses were that (1) the PLS-SEM could create a composite index to measure the overall players’ physical performance, but (2) individual playing time and position would be influential features due to significant correlations between time and performance as well as differences between playing positions.

## Methods

### Study design

This is an observational and retrospective study which includes a total of 830 official matches that were analyzed. Data were collected from 42 professional soccer teams participating in LaLiga 2021–22 Men’s First Division and Spanish Copa del Rey. The physical performance variables were collected using EPTS. Each match had a duration of 90 min plus additional time. Six matches included an overtime period of 30 min as they were part of Copa del Rey.

### Participants

A total of 24,980 match observations were collected from 42 teams, including 1138 male professional soccer players. Each player was categorized based on the following positions: forwards (n = 286), midfielders (n = 441), and defenders (n = 411). The players were included in the study if they participated either in the Spanish Men’s First Division League or Spanish Copa del Rey in the 2021/2022 season. All players’ performance data were considered for this study (including any substitutions and the extra-time period from Copa del Rey matches). Due to the different nature of their activity profile, goalkeepers were not included in the study. All the information was sourced from LaLiga, which permitted the examination of variables investigated in this study and the dissemination of results with a scientific aim. Adhering to LaLiga’s ethical standards, this study abstains from disclosing any data that could identify individual soccer players. We confirm that all methods were carried out in accordance with relevant guidelines and regulations; in particular, all experimental protocols were approved by LaLiga (www.laliga.com); subsequently, informed consent was obtained from all subjects. LaLiga granted permission for the use of these data in this investigation, which received approval from the Institutional Review Board.

### Procedures

Performance data were gathered using the computerized multi-camera tracking system TRACAB Gen4 (ChyronHego, New York, USA), which is a recognized technology for soccer-specific performance analysis^20^. TRACAB’s tracking systems are deemed valid technologies for soccer-specific performance analyses^20^. This system recorded positioning and motion data through a computerized multi-camera approach. Subsequently, a customized report was generated with the assistance of Mediacoach software (www.mediacoach.es, LaLiga, Madrid, Spain). This software synchronized the tracking data with video footage of each match. Also, to ensure data accuracy, a quality control process was implemented by Mediacoach after each match. This process involved cross-referencing the TRACAB data with TRACAB’s own algorithm and conducting a player-by-player review to rectify any potential errors inherent in the optical tracking technology. This meticulous quality control procedure not only enhances the quality of the data but also enables professionals to visualize and analyze the performance tracking data, as outlined in the work of Refs.^21,22^.

Specifically, the physical performance variables from the Mediacoach report (LaLiga, Madrid, Spain) powered by WIMU (Realtrack Systems, Almería, Spain)^2^ were used. This report can be accessed via the Mediacoach portal (www.portal.mediacoach.es) by clicking on the integrated WIMU app. Therefore, the following variables were included in the study: total distance (m), explosive distance (distance covered in meters with a given acceleration, e.g., greater 1.12 m/s^2^), distance covered (m) by speed zone (i.e., ≤ 6 km/h; 6–12 km/h; 12–18 km/h; 18–21 km/h; 21–24 km/h; > 24 km/h), time spent (ms) and count of actions by speed zone, total of accelerations (count), total of decelerations (count), maximum acceleration (m/s^2^), maximum deceleration (m/s^2^), average acceleration (m/s^2^), average deceleration (m/s^2^), distance in acceleration (m), distance in deceleration (m), total of accelerations by zone (i.e., ≤ 1 m/s^2^; 1–2 m/s^2^; 2–3 m/s^2^; 3–4 m/s^2^; 4–5 m/s^2^; 5–6 m/s^2^; > 6 m/s^2^), total of decelerations (i.e., negative acceleration value) by zone (≤ 1 m/s^2^; 1–2 m/s^2^; 2–3 m/s^2^; 3–4 m/s^2^; 4–5 m/s^2^; 5–6 m/s^2^; > 6 m/s^2^), distance covered by acceleration zone, distance covered by deceleration zone, total of time (ms) by acceleration zone, total of time (ms) by deceleration zone, sprint duration (s), total of sprints (above 24 km/h), total of high speed running actions (above 21 km/h), sprinting distance (m, above 24 km/h), high-speed running distance (m, above 21 km/h), maximum speed (km/h), average speed (km/h), total power metabolic (W/kg), mean power metabolic (W/kg), maximum equivalent distance index, high-metabolic load distance (m), high-metabolic load actions (count), and energy expenditure (kcal).

### Statistical analysis

To create the composite index and measure the overall players’ physical performance in professional soccer matches, the dataset was filtered in the following way. Firstly, a correlation analysis was conducted to remove variables because of collinearity problems. Then, a total of 36 variables were selected. Specifically: total distance, explosive distance, total of actions by speed zone (6–12 km/h; 12–18 km/h; 18–21 km/h; 21–24 km/h; > 24 km/h), time spent (ms) by speed zone (6–12 km/h; 12–18 km/h; 18–21 km/h; 21–24 km/h; > 24 km/h), total of accelerations by each zone (≤ 1 m/s^2^; 1–2 m/s^2^; 2–3 m/s^2^; 3–4 m/s^2^; 4–5 m/s^2^; 5–6 m/s^2^; > 6 m/s^2^), total of decelerations by each zone (≤ 1 m/s^2^; 1–2 m/s^2^; 2–3 m/s^2^; 3–4 m/s^2^; 4–5 m/s^2^; 5–6 m/s^2^; > 6 m/s^2^), average acceleration, average deceleration, maximum speed, average speed, total power metabolic, mean power metabolic, maximum equivalent distance index, high-metabolic load distance, count of high-metabolic load actions, and energy expenditure (kcal).

Secondly, a Principal Component Analysis (PCA)^23^ was conducted as explorative analysis and for detecting some latent factors behind the physical performance composite index and select the most related variables. Specifically, three significant latent components were found (eigenvalues greater than 1); those explained at about the 95% of the initial variability. At this point, for each latent component the most important (i.e., with loading factor |λ| > 0.65) variables were selected after a varimax rotation. Then, 17 variables, which were strictly related to their own components (Table 1), were selected.

**Table 1:** Output of the principal component analysis.

Component	Compact name	Variable
Component 1	C1_A	Count of accelerations (zone 2–3 m/s^2^)
Component 1	C1_B	Count of accelerations (zone 3–4 m/s^2^)
Component 1	C1_C	Count of accelerations (zone 4–5 m/s^2^)
Component 1	C1_D	Count of accelerations (zone 5–6 m/s^2^)
Component 1	C1_E	Count of decelerations (zone 2–3 m/s^2^)
Component 1	C1_F	Explosive distance
Component1	C1_G	Count of actions (zone 6–12 km/h)
Component 2	C2_A	Count of actions (21–24 km/h)
Component 2	C2_B	Count of actions (> 24 km/h)
Component 2	C2_C	Time spent (zone 21–24 km/h)
Component 3	C3_A	Average speed (km/h)
Component 3	C3_B	Count of actions (zone 12–18 km/h)
Component 3	C3_C	Count of actions (zone 18–21 km/h)
Component 3	C3_D	Time spent (zone 18–21 km/h)
Component 3	C3_E	Energy expenditure
Component 3	C3_F	High-metabolic load actions
Component 3	C3_G	High-metabolic load distance

Then, we decided to adopt a non-parametric approach for creating the composite indicator, the PLS-SEM approach, a trend-method many used in social sciences^15^. In particular, a hierarchical (Second Order) PLS-SEM algorithm was completed using the smartPLS software (www.smartpls.com, version 3.3.7) and the R package seminR (version 2.3.2, with 5000 bootstrap resampling)^24^ by a Mixed Two-Step approach^25^ to estimate the Second Order construct^12^. Finally, as player index adjusment phase, a normalization process was made for each player their own indices time series and then the index was translated in a clearer evaluation scale (between 0 and 10), taking into account 130 min as maximum target for the players’ physical performance (e.g., considering matches with 30 min overtime and the extra time). A detailed procedure of the formulas applied at this stage were provided in the results section given the nature of this study.

Once the composite index was created, a linear regression analysis was carried out to explore relationships between playing activity time and the composite index. In addition, the Kruskal–Wallis test was conducted to analyze the differences in the composite index between playing positions. Effect sizes were calculated through eta squared. A larger eta squared value indicated a stronger effect of the independent variable(s) on the dependent variable while a value closer to 0 suggested a smaller effect size. Specifically, the effect sizes were interpreted as follows: small effect size (Eta squared ≤ 0.01), medium effect size (0.01 < Eta-squared ≤ 0.06), and large effect size (Eta-squared > 0.06)^26^.

## Results

### Composite index

**Figure 1:**
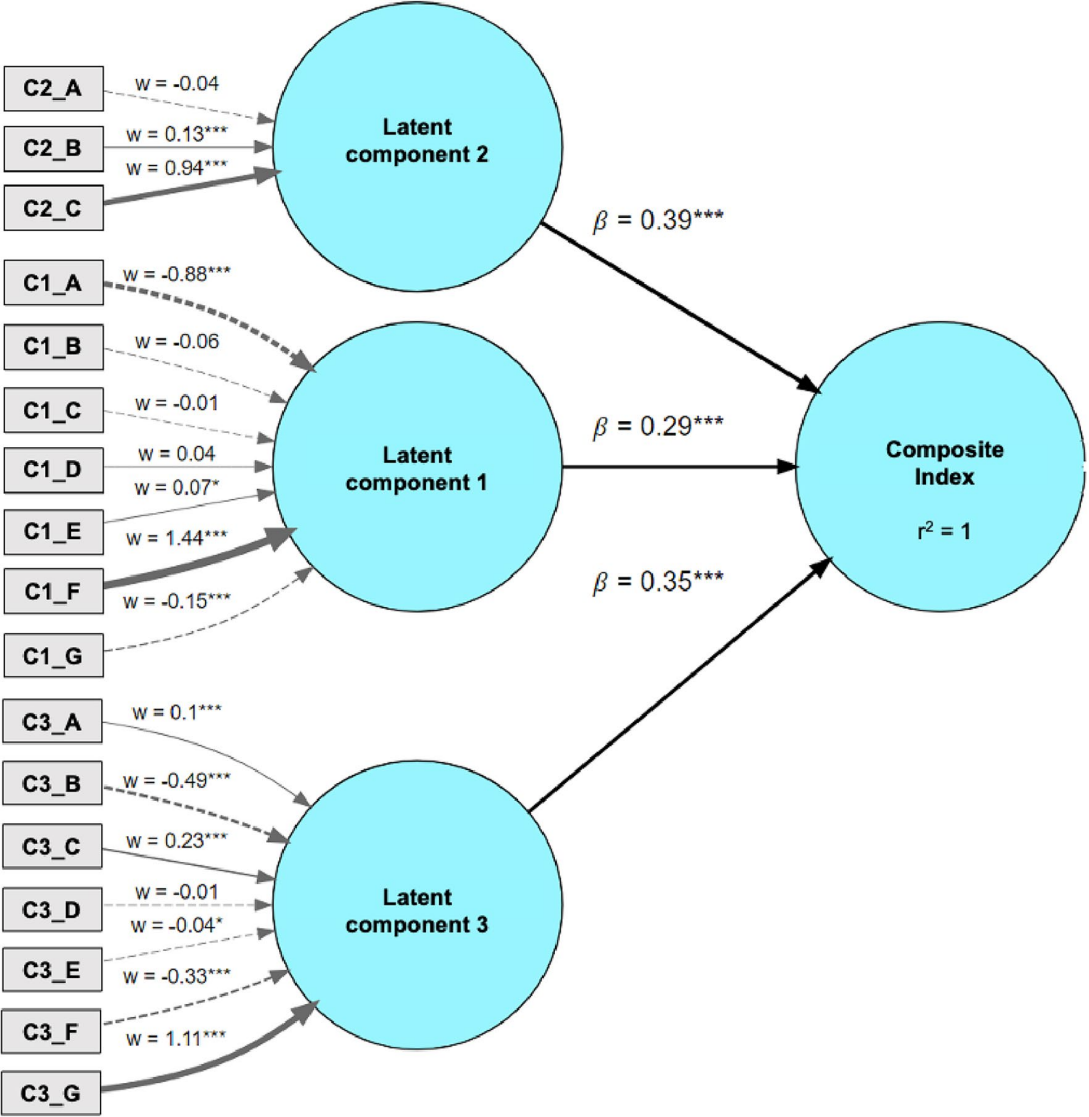
The PLS-SEM output.

Figure 1 shows the PLS-SEM output. Specifically, three significant latent components were found, which explained 95% of the initial variability and were related to the acceleration-specific performance (component 1), high-intensity running-related variables (component 2), and medium intensity actions (component 3). From a practical point of view, to compute the composite index for a generic player *i* and given its set of 17 physical performance variables, the following process needs to be followed (based on the weights *w* of Fig. 1):***

1) First of all, for each player *i* the composite index needs to be computed for each lower order component using formula (1–3):

1 $${Latent\;component\;1}_{i}=-0.88*{C1\_A}_{i}-0.06*{C1\_B}_{i}-0.01*{C1\_C}_{i}+0.04*{C1\_D}_{i}+0.07*{C1\_E}_{i}+1.44*{C1\_F}_{i} -0.15*{C1\_G}_{i}$$

2 $${Latent\;component\;2}_{i}=-0.04*{C2\_A}_{i}+0.13*{C2\_B}_{i}+0.94*{C2\_C}_{i}$$

3 $${Latent\;component\;3}_{i}=0.10*{C3\_A}_{i}-0.49*{C3\_B}_{i} +0.23*{C3\_C}_{i} - 0.01*{C3\_D}_{i}-0.04*{C3\_E}_{i}-0.33*{C3\_F}_{i}+1.11*{C3\_G}_{i}$$

2) Then, to compute the raw composite index rough for each player, apply formula (4):

4 $${Raw\;composite\;index}_{i}=0.29*{Latent\;component\;1}_{i}+0.39*{Latent\;component\;2}_{i}+0.35*{Latent\;component\;3}_{i}$$

3) Finally, to obtain the normalized composite index in a 10-point scale (0 being the lowest performance and 10 being the highest) for an easy interpretation of the index, formula (5) should be used for each player *i*.

5 $${Composite\;index}_{i}=\frac{10*({Raw\;composite\;index}_{i}-\text{min}({Raw\;composite\;index}_{i}))}{\text{max}({Raw\;composite\;index}_{i})-\text{min}({Raw\;composite\;index}_{i})}$$

where $\text{max}({Raw\;composite\;index}_{i})$ and $\text{min}(Raw\;{composite\;index}_{i})$ are respectively the minumum and maximum raw composite index of the series values (i.e., considering all the players).

### Effect of playing time and position

**Figure 2:**
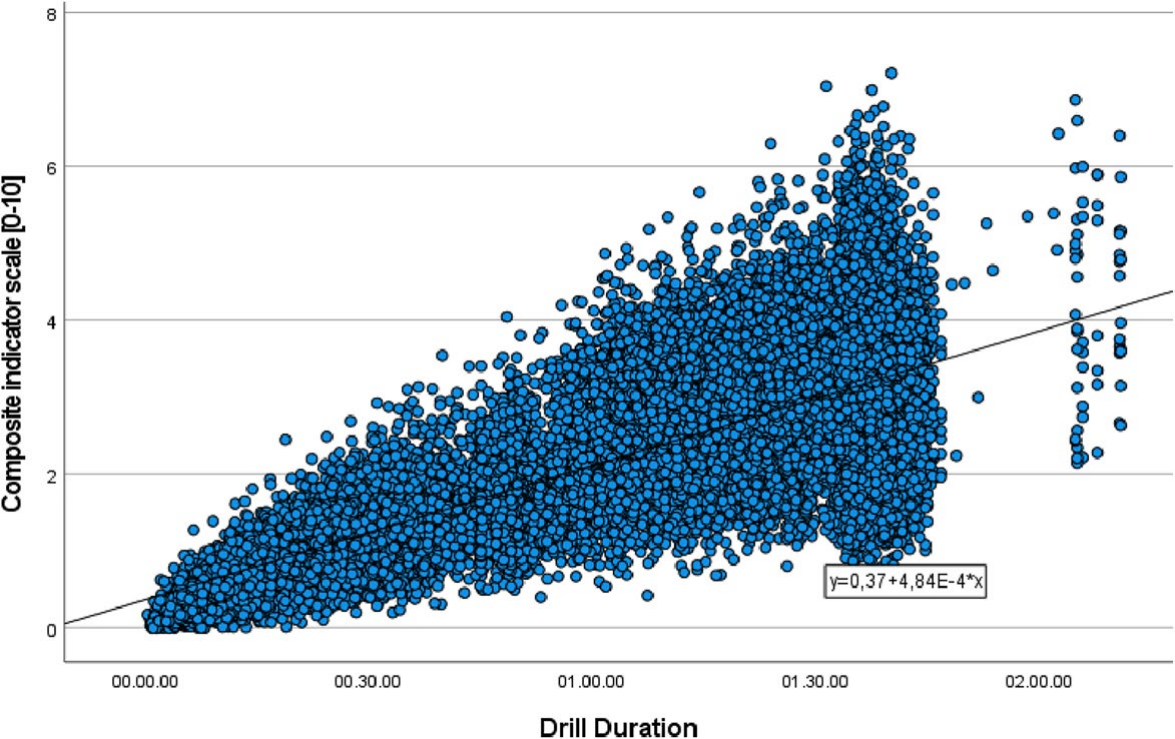
Linear regression analysis to explore relationships between playing activity time (hours—X axis) and the composite index (10-point scale—Y axis).

Figure 2 shows a strong and positive correlation between individual playing time and the composite index (*r* = 0.76; *p* < 0.001; R^2^ = 0.58). In addition, significant positive correlations were observed in forwards (*r* = 0.85; *p* < 0.001; R^2^ = 0.74), midfielders (*r* = 0.80; *p* < 0.001; R^2^ = 0.64), and defenders (*r* = 0.67; *p* < 0.001; R^2^ = 0.45). Also, the results showed significant differences between playing positions with small effect size (*p* < 0.05; eta-squared = 0.01).

## Discussion

The purpose of this study was to create a composite index to measure the overall players’ physical performance in professional soccer matches and analyze the effect of individual playing time and positional differences on this composite index. The novelty of this study was that this method could reduce all the information collected in the physical performance report to one single variable. Specifically, three significant latent components were found, which explained 95% of the initial variability, that were related to the acceleration-specific performance (component 1), high-intensity running-related variables (component 2), and medium intensity actions variables (component 3). Also, a strong and positive correlation was observed between individual playing time and the composite index, but positional differences may be observed.

The physical performance composite index which was created in this study is a very novel approach for the assessment of physical performance. This computational approach, which is based on the PLS-SEM hierarchical model, is an original application in the sport field^12^ and has never been applied to professional soccer players performance data, to the best of our knowledge. Previous studies have applied other statistical methods to reduce the number of physical performance variables from their reports^2,27^. For instance, a recent study explained that 7 variables, which included: metabolic power, total of steps, Fourier transform duration, deceleration distance covered (2–3 m/s^2^), total of running actions (12–18 km/h; 21–24 km/h) were the selected variables, belonged to the first two components of the PCA and explained 80% of total variance^2^. In this regard, another study found three components in the PCA that represented the ~ 59% of total variance (component 1: distance per minute, explosive distance, distance per minute in zones like 18–21 km/h and 21–24 km/h; component 2: accelerations and decelerations; component 3: maximum acceleration and deceleration)^27^. In this regard, there is a level of similarity between the types of variables that were found as important parameters to analyze (e.g., mid-intensity and high-intensity running actions: average speed or meters per minute, and actions above 18, 21, or 24 km/h; variables with accelerations and deceleration component: explosive distance, total of accelerations/decelerations and considering different speed bands) and they may explain the importance of understanding soccer as a sport characterized by high-intensity actions interspersed with longer recovery periods of lower intensity^2,28^.

Furthermore, strong, and positive correlations were observed between individual playing time and the composite index, but positional differences may be observed. The fact that there is a positive linear relation in relation to time was expected because of the natural evolution of the match demands as the players stay on the field^18^. However, this was important to analyze to gain a better understanding of how the physical performance composite index that was created would change throughout the course of match-play. In addition, positional differences were observed and these were in line with the initial hypothesis. Multiple studies have shown that physical performance is dependent not only on playing position, but also on other contextual factors (e.g., team formation, ball in play, competitive standards, match status, etc.)^29–31^, so future research could be done in order to gain a better understanding of how these contextual variables impact the physical performance composite index.

However, this study has some limitations. For example, the physical performance data were collected from video-tracking systems so no information about the physiological response of the players (e.g., mean heart rate, time spent in different heart rate zones, etc.) was provided. Although future research is required in order to explore the applicability of these methods to pinpoint and select key performance indicators, it is necessary to ensure that data quality from the original performance reports is examined. Also, the playing positions were categorized in three groups while there could be a more extended approach based on various team formations. In this regard, future studies could consider specific positions such as central defenders, full-backs, central midfielders, wide-midfielders, and forwards^18,19^. Finally, another limitation was that only 6 matches were included in the analysis of matches with overtime periods, which is imbalanced in comparison with the total of match observations from regular 90-min matches.

## Practical implications

This study may serve as a reference for sports performance practitioners to create a composite index that measures the overall players’ physical performance, so the instructions to create it are available in the manuscript. In addition, this composite index may be used for correlation with technical-tactical parameters, which may be an opportunity to understand the weight of physical output on individual and/or team performance. Future research is necessary to have a better understanding of the applicability of this data reduction method not only in professional soccer but in other sports. In addition, given the importance of the variables the contributed to the three main components of the PLS-SEM output, coaches may consider the variables from Table 1 the analysis of physical performance.

## Data Availability

The datasets used and/or analysed during the current study available from the corresponding author on reasonable request. LaLiga granted permission for the use of these data in this investigation, which received approval from the Institutional Review Board.

## References

[1] Bandyopadhyay K (2017). Legacies of Great Men in World Soccer.

[2] Oliva-Lozano JM, Barbier X, Fortes V, Muyor JM (2021). Key load indicators and load variability in professional soccer players: A full season study. Res. Sports Med. [Internet]..

[3] Chmura P, Oliva-Lozano JM, Muyor JM, Andrzejewski M, Chmura J, Czarniecki S, Kowalczuk E, Rokita A, Konefał M (2022). Physical performance indicators and team success in the German soccer. J. Hum. Kinet. [Internet]..

[4] Anderson L, Orme P, Di Michele R, Close GL, Morgans R, Drust B, Morton JP (2016). Quantification of training load during one-, two- and three-game week schedules in professional soccer players from the English Premier League: Implications for carbohydrate periodisation. J. Sports Sci. [Internet]..

[5] Oliveira R, Brito J, Martins A, Mendes B, Calvete F, Carriço S, Ferraz R, Marques MC (2019). In-season training load quantification of one-, two- and three-game week schedules in a top European professional soccer team. Physiol. Behav..

[6] Oliveira R, Brito JP (2023). Load monitoring and its relationship with healthcare in sports. Healthcare..

[7] Torres-Ronda L, Beanland E, Whitehead S, Sweeting A, Clubb J (2022). Tracking systems in team sports: A narrative review of applications of the data and sport specific analysis. Sports Med. Open [Internet]..

[8] Pino-Ortega, J., Oliva-Lozano, J.M., Gantois, P., Nakamura, F.Y., Rico-González, M. (2021) Comparison of the validity and reliability of local positioning systems against other tracking technologies in team sport: A systematic review. *Proc. Inst. Mech. Eng. P. J. Sport Eng. Technol*. 10.1177/1754337120988236

[9] Oliva-Lozano JM, Martín-Fuentes I, Granero-Gil P, Muyor JM (2022). Monitoring elite soccer players physical performance using real-time data generated by electronic performance and tracking systems. J. Strength Cond. Res. [Internet]..

[10] Rojas-Valverde D, Gómez-Carmona CD, Gutiérrez-Vargas R, Pino-Ortega J (2019). From big data mining to technical sport reports: The case of inertial measurement units. BMJ Open Sport Exerc. Med..

[11] Rojas-Valverde D, Pino-Ortega J, Gómez-Carmona CD, Rico-González M (2020). A systematic review of methods and criteria standard proposal for the use of principal component analysis in team’s sports science. Int. J. Environ. Res. Public Health..

[12] Cefis M, Carpita M (2022). The higher-order PLS-SEM confirmatory approach for composite indicators of football performance quality. Comput. Stat..

[13] Carpita M, Pasca P, Arima S, Ciavolino E (2023). Clustering of variables methods and measurement models for soccer players’ performances. Ann. Oper. Res..

[14] Field A, Naughton RJ, Haines M, Lui S, Corr LD, Russell M, Page RM, Harper LD (2022). The demands of the extra-time period of soccer: A systematic review. J. Sport Health Sci..

[15] Lago-Peñas C (2012). The influence of effective playing time on physical demands of elite soccer players. Open Sports Sci. J..

[16] Liu, H., Wang, L., Huang, G., Zhang, H., Mao, W. Activity profiles of full-match and substitution players in the 2018 FIFA World Cup. *Eur. J. Sport Sci*. 1–7 (2019). 10.1080/17461391.2019.1659420 31495296

[17] Dalen T, Lorås H (2019). Monitoring training and match physical load in junior soccer players: Starters versus substitutes. Sports..

[18] Oliva-Lozano JM, Granero-Gil P, Panascì M (2023). Changes in physical performance throughout professional soccer match-play. J. Strength Cond. Res..

[19] Casamichana D, Martín-García A, Gómez Díaz A, Bradley S, P, Castellano J, (2022). Accumulative weekly load in a professional football team: With special reference to match playing time and game position. Biol. Sport..

[20] Linke D, Link D, Lames M (2020). Football-specific validity of TRACAB’s optical video tracking systems. Kerhervé HA, editor. PLoS One..

[21] Pons, E., García-Calvo, T., Resta, R., Blanco, H., López del Campo, R., Díaz García, J., Pulido, J.J. A comparison of a GPS device and a multi-camera video technology during official soccer matches: Agreement between systems. Sunderland C, editor. *PLoS One*. **14**(8):1–12 (2019). 10.1371/journal.pone.0220729 10.1371/journal.pone.0220729 PMC6687125 31393932

[22] Felipe JL, Garcia-Unanue J, Viejo-Romero D, Navandar A, Sánchez-Sánchez J (2019). Validation of a video-based performance analysis system (Mediacoach®) to analyze the physical demands during matches in LaLiga. Sensors..

[23] Abdi H, Williams LJ (2010). Principal component analysis. WIREs Comput. Stat..

[24] Shmueli G, Ray S, Velasquez Estrada JM, Chatla SB (2016). The elephant in the room: Predictive performance of PLS models. J. Bus Res..

[25] Crocetta C, Antonucci L, Cataldo R, Galasso R, Grassia MG, Lauro CN, Marino M (2021). Higher-order PLS-PM approach for different types of constructs. Soc. Indic. Res..

[26] Cohen J. Statistical power analysis for the behavioral sciences [Internet]. 2nd ed. Hillsdale N.J.: L. Erlbaum Associates; 1988 [cited 2019 Mar 2]. 567 p. Available https://www.worldcat.org/title/statistical-power-analysis-for-the-behavioral-sciences/oclc/17877467

[27] Rojas-Valverde D, Gómez-Carmona CD, Bastida Castillo A, Nakamura FY, Giménez-Martínez E, Matabosch-Pijuán M, Bernal JR, Pino-Ortega J (2023). A longitudinal analysis and data mining of the most representative external workload indicators of the whole elite Mexican soccer clubs. Int. J. Perform. Anal. Sport..

[28] Varley M, Aughey R (2012). Acceleration profiles in elite Australian soccer. Int. J. Sports Med..

[29] Aquino R, Vieira LHP, Carling C, Martins GHM, Alves IS, Puggina EF (2017). Effects of competitive standard, team formation and playing position on match running performance of Brazilian professional soccer players. Int. J. Perform. Anal. Sport..

[30] Riboli A, Semeria M, Coratella G, Esposito F (2021). Effect of formation, ball in play and ball possession on peak demands in elite soccer. Biol. Sport..

[31] Zhou C, Hopkins WG, Mao W, Calvo AL, Liu H (2019). Match performance of soccer teams in the Chinese Super League—Effects of situational and environmental factors. Int. J. Environ. Res. Public Health..

